# A genomic biomarker for the rapid identification of the rob(1;29) translocation in beef cattle breeds

**DOI:** 10.1038/s41598-024-53232-8

**Published:** 2024-02-05

**Authors:** Alessandra Iannuzzi, Sebastián Demyda-Peyrás, Ramona Pistucci, Rosa Morales, Michele Zannotti, Fiorella Sbarra, Andrea Quaglia, Pietro Parma

**Affiliations:** 1 Institute for Animal Production System in Mediterranean Environment, National Research Council, 80055 Portici, Italy; 2 Departamento de Genética, Universidad de Córdoba, Campus Rabanales, 14014 Córdoba, Spain; 3 Facultad de Ciencias Veterinarias, Universidad Nacionald E La Plata, 1900 La Plata, Argentina; 4 Department of Agricultural and Environmental Sciences, University of Milan, 20133 Milan, Italy; 5 National Association of Italian Beef-Cattle Breeders (ANABIC), 06132 San Martino in Colle, Perugia Italy

**Keywords:** Genetics, Cytogenetics

## Abstract

Robertsonian translocations, specifically rob(1;29) translocation, have reportedly been the most prevalent chromosomal abnormalities in cattle, affecting various breeds and leading to a decrease in fertility and reproductive value. Currently, the identification of rob(1;29) carriers relies on cytogenetic analysis that has limitations in terms of accessibility, cost, and sample requirements. To address these limitations, a novel genomic biomarker was developed in this study for the rapid and precise identification of rob(1;29) carriers. Using q-PCR, a specific copy number variation associated with translocation was targeted, which effectively distinguished between wild-type, homozygous and heterozygous carriers. Crucially, the biomarker can be applied to DNA extracted from various biological matrices, such as semen, embryos, oocytes, milk, saliva, coat, and muscle, and it is compatible with fresh, refrigerated, or frozen samples. Furthermore, this approach offers significant reductions in cost compared to those associated with traditional cytogenetic analysis and provides results within a short turnaround time. The successful development of this genomic biomarker has considerable potential for widespread adoption in screening programs. It facilitates timely identification and management of rob(1;29) carriers while mitigating economic losses and preserving genetic integrity in bovine populations.

## Introduction

**Figure 1:**
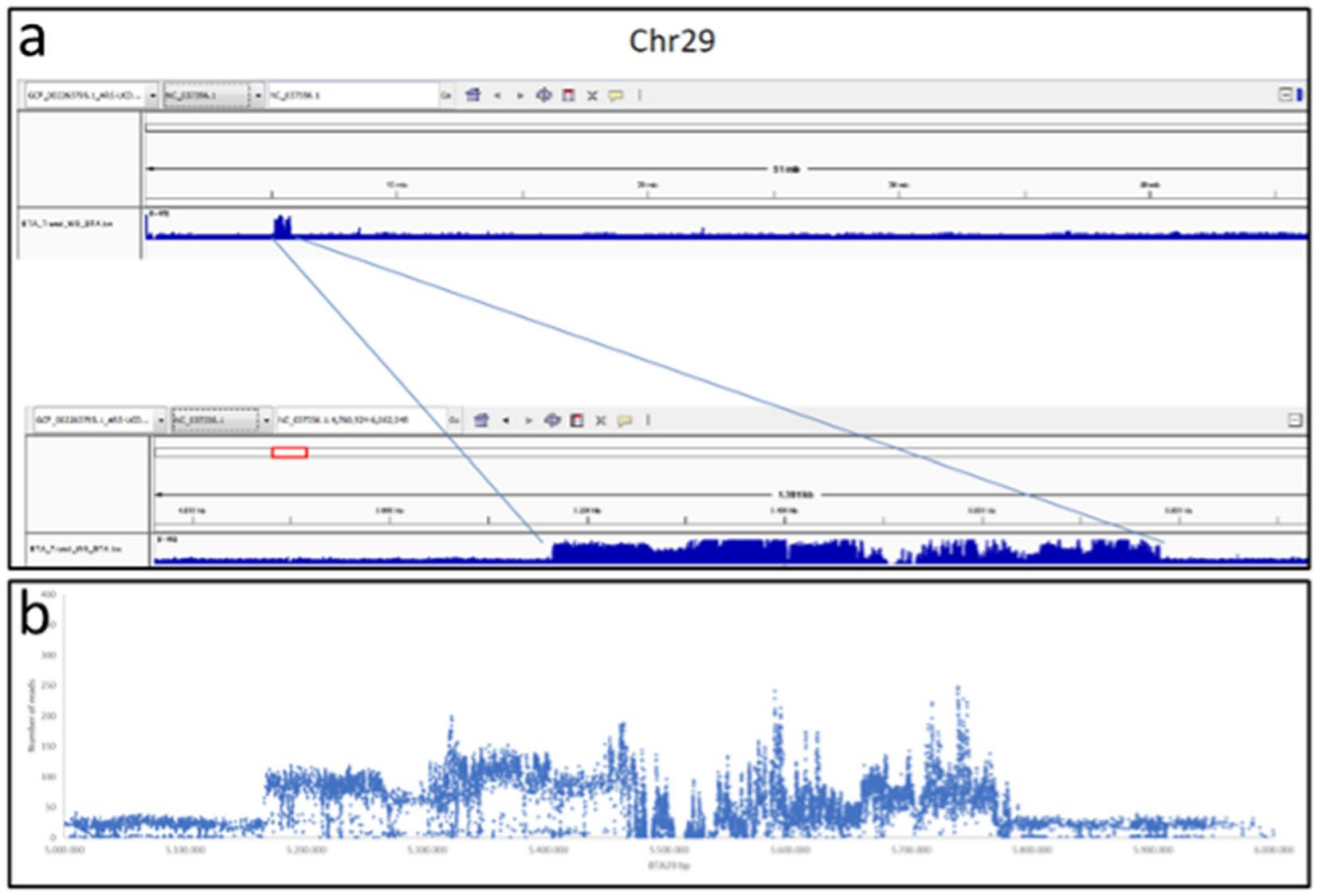
The Integrative Genomic Viewer (IGV) visualization of the rob(1;29) specific CNV: (**a**) Display of the region containing the specific CNV; (**b**) Analysis of the number of reads sequenced in the presence of SNPs contained in the region of interest.

Robertsonian or centric-fusion (CF) translocations are the most commonly found chromosome abnormalities in cattle (Bos taurus) due to the break and fusion of two acrocentric chromosomes in the centromeric region. Among these, Robertsonian translocation 1;29 (rob1;29) (Fig. 1S), first reported by Gustavsson and Rockborn^1^, is the most widespread and prevalent in across 50 different cattle breeds^2^. Field studies have reported an incidence of rob(1;29) in various cattle breeds ranging from 1 to 70%. It causes a decrease in fertility, ranging from 9 to 18% (depending on gender carrier), and reproductive value of carriers (approximately 8–9%)^3–8^. This phenomenon is attributed to the formation of unbalanced gametes, an anomaly that leads to the development of nonviable embryos that cannot survive beyond the ninth day of development^9,10^. All identified rob(1;29) carriers to date have inherited this anomaly from one of their parents, suggesting the existence of a common ancestor from whom the translocations spread. This conclusion derives from several facts: (a) To date, no report on rob(1;29) de novo has been published, despite the known fact that this would be very important information. If this identification were made possible in the past, it would have offered the possibility of observing rob(1;29) in its initial form, probably dicentric, without inversion of the centromeric region of BTA29. (b) In our laboratory alone (Milano University, P. Parma, personal communication), more than 1200 carriers have been identified, with none of them being de novo.

**Figure 2:**
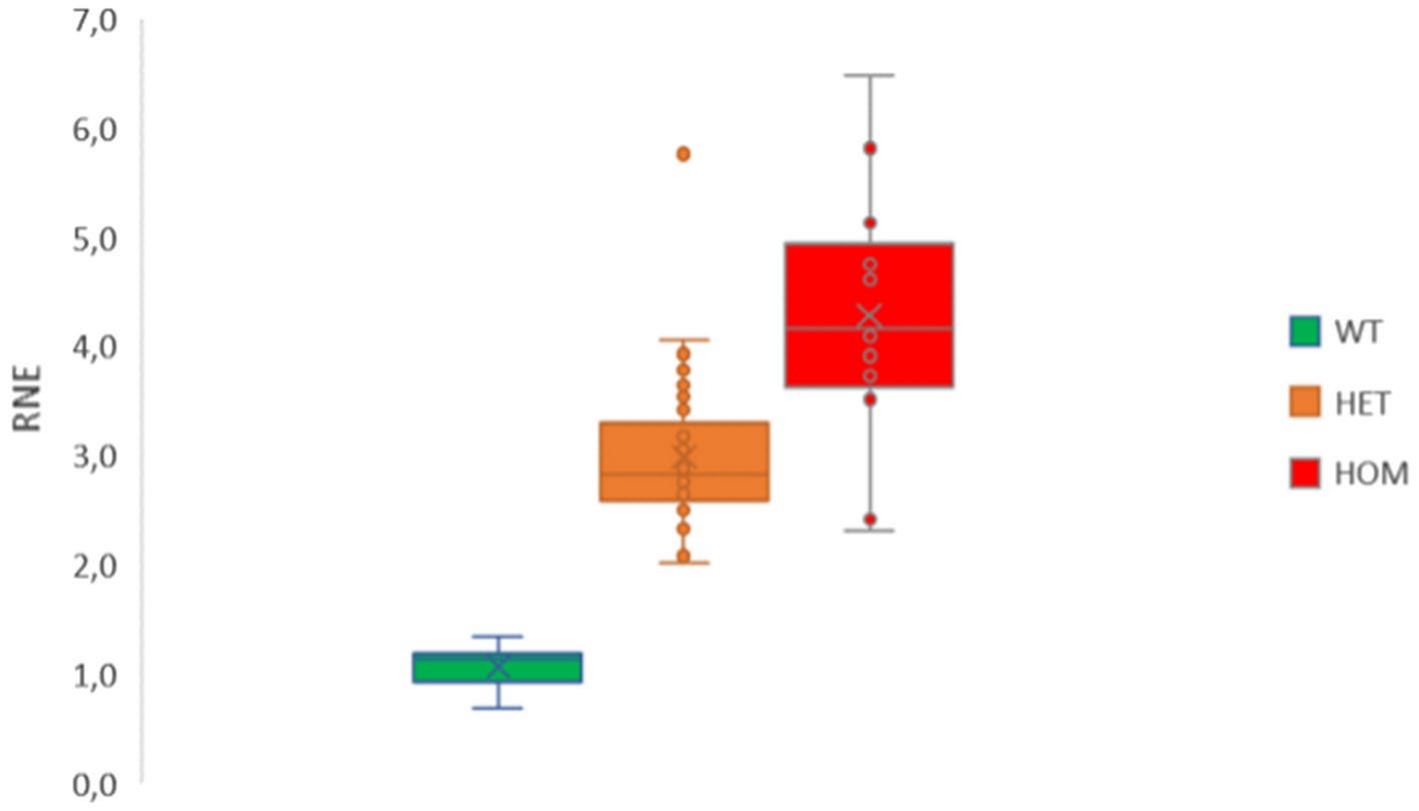
Bar chart of average Relative Normalized expression (RNE) values for each sample. The samples are categorized as follow: Wild type (Wt), Heterozygous carriers (HET), and Homozygous carriers (HOM). Statistical significance is denoted by different letters with a significance level of p < 0.001.

Interestingly, the prevalence of rob(1;29) appears to be higher in breeds intended for meat production, particularly in Barrosa (Portugal), where it can reach 70%^11–13^. However, it has also been found in breeds for milk production^14^. To mitigate the detrimental effects of rob(1;29) and the associated economic losses, several countries have implemented early screening programs since the early 1990s^14^. Notably, Italy and Spain have demonstrated remarkable commitment to rob(1;29) screening, particularly in the Chianina, Marchigiana, Maremmana, Romagnola, Podolica, and Retinta cattle breeds. In Italy, the animal cytogenetics laboratory of the University of Milan has played a leading role in its screening program, having tested over 21,000 animals since 1993 (Table 1S; Parma, personal communication). Consequently, the prevalence of carrier subjects decreased considerably across all breeds. However, recent trends indicate a potential slowdown in eradication (Fig. 2S) due to a reduction in the number of animals tested for rob(1;29), primarily due to the high cost associated with the analysis. Currently, the identification of rob(1;29) carriers heavily relies on cytogenetic analyses that use traditional techniques with limited advancements^15^. Nevertheless, this approach has a significant limitation: the requirement for fresh blood samples to reach the laboratory within a narrow timeframe of 24–48 h. Other main limitations are the waiting time (7–10 days) and higher costs associated with the materials and qualified personnel. These stringent requirements restrict the feasibility of testing animals in remote locations or situations in which obtaining a blood sample is challenging. Vozdova et al. identified a specific chromosomal marker for rob(1;29) that allows detection only in bovine spermatozoa^16^. However, this detection technology involves more complex fluorescence in situ hybridization (FISH) analysis compared to classic cytogenetic methods^17^.

To overcome these limitations, this study proposes a novel genomic biomarker for the identification of rob(1;29) carriers in Italian and Spanish cattle breeds, distinguishing between wild-type normal subjects (Wt), heterozygous (HET) and homozygous (HOM) carriers through rapid, low-cost, and specific DNA analysis. By leveraging this new approach, this study aims to provide a more accessible and efficient screening method for rob(1;29) translocation while overcoming the constraints associated with traditional cytogenetic methods.

## Results

### CNV analysis

Genomic sequencing generated 323,091,667 reads, resulting in an average genomic coverage of 15 × . To identify a genomic region specific to the rob(1;29) chromosome, the focus of this study was on the first 10 Mb of BTA29. This region is known to undergo minimal or no recombination during the meiotic process. One overrepresented region was effectively present around 5.4 Mb of the BTA29, as shown by the study of the coverage acquired after genomic sequencing (Fig. 1a). This region was 615 Kb long and spanned the genomic area between bases 5,165,268 and 5,780,610 of BTA29 (ARS-UCD-1.3 cattle genome assembly). Regarding the whole BTA29, the median number of reads coverage is equal to 21 × , while in the region concerned, this value is 65 × (Fig. 1b).

In this genomic region, 33 genetic factors found in the NCBI Release106 and Ensemble Release 95 databases, were included (Table 2S). Of these factors, three belonged to the TRIM gene family—genetic factors that contain a tripartite motif (TRIM) ubiquitin ligase—that acts in the innate immune response against viruses^18^. This region contained 12,551 SNPs (an average of 1 every 49 bases), and 2,242 of these were present in the homozygous state. In 75% of heterozygous SNPs, the alternative variant was preferred to the reference variant, and on average, the alternate variant was present in 73% of the reads. SNPs in which the reference variant was the most represented were found in the centromeric region of CNV (Fig. S3).

This region seemed to be a great candidate specifically for rob(1;29) examined in this study by q-PCR, the copy numbers of this region in normal subjects, heterozygous carriers, and homozygous carriers.

### q-PCR efficiency

To assess the q-PCR amplification efficiency, a standard curve was prepared. This involved preparing four concentrations of cattle genomic DNA obtained from a mix of four samples using fourfold serial dilutions. These diluted DNA samples were then aliquoted in triplicates on the q-PCR plate. Two independent standard curves were generated for the T and SCG sequences. The SYBR Green fluorescence signal was acquired at two different temperatures: 77 °C for the target sequence and 87 °C for the SCG sequence in each cycle (Fig. 4S). The q-PCR efficiency (E) was 97.5% for the target primer and 99.6% for the SCG primer. All samples included in the analysis presented a standard deviation (SD) of less than 0.2. The relative normalized expression (RNE) was calculated after measuring the ratio of T repeats to SCG (T/S ratio) for each sample using the ΔΔCt method.

### CNV using qPCR analysis

The results revealed that the T sequence was overrepresented in all carriers, distinguishing between Wt subjects, HOM and HET carriers (Fig. 2). Interestingly, it is noteworthy that the RNE confidence interval values did not overlap, suggesting the excellent genotype identification capability of the subjects. Furthermore, the separation between controls (Wt) and HET carriers was greater than that between HET and HOM, as expected. The RNE values obtained for the three genotypes were significantly different (p < 0.001) (Fig. 2, Table 1).

**Table 1:** Statistic for Relative Normalized Expression (RNE) values within each of the three genotypes in the population study: wild type (Wt), Heterozygous carriers (HET), and Homozygous carriers (HOM). Mean (AVG), standard deviation (SD) and 99% confidence intervals (IC) of the RNE value are reported for each genotype.

	Avg	SD	N	CI 0.999	
Wt/Wt	1.069	0.199	14	0.844	1.294
Wt/rob	2.951	0.713	54	2.613	3.289
rob/rob	4.286	1.186	13	2.866	5.706

## Discussion

To identify a specific genomic region associated with rob(1;29) chromosome translocation, this study focused on the centromeric region of the BTA29 chromosome. This region is known to undergo minimal or no recombination during the meiotic process, as observed in previous studies on synaptonemal complexes^19^. This characteristic was further confirmed through analysis of the genomic structure of rob(1;29)^20^. The translocation of this region from the centromeric region of BTA29 to the centromeric region of the q-arm of rob(1;29) prevents the formation of synaptonemal complexes and inhibits recombination. Based on the hypothesis suggesting a common ancestor among all rob(1;29) carriers, this region represents the most likely candidate for ensuring the presence of a specific haplotype associated with BTA rob(1;29). To determine the specificity of this region for rob(1;29), q-PCR analysis was performed to assess CNVs in Wt subjects, HET carriers, and HOM carriers. The results confirmed that this CNV was indeed specific to the rob(1;29).

This approach could also be used for other anomalies, provided that anomaly-specific genomic regions are available. However, since the bovine species rob(1;29) is the only recurrent abnormality in the current situation, no alternative seems to exist for cytogenetic analysis to identify other abnormalities.

The proposed genomic biomarker has broad applicability beyond blood samples. It can be utilized on various bovine biological matrices, including semen, embryos, oocytes, milk, saliva, coat, and muscle, as long as DNA extraction is possible. In addition, the biomarker can be applied to fresh, refrigerated (at 4 °C), or even frozen (at − 20 °C) samples that have been stored for an extended period. This versatility allows for the convenient and efficient screening of a wide range of samples, regardless of their storage conditions. Its feasibility in analyzing different biological matrices and accommodating various storage conditions enhances the practicality and applicability of the proposed method in diverse settings, making it a valuable diagnostic tool.

One important advantage of this approach is its cost-effectiveness, with each analysis estimated to have a 90% reduction in cost compared to those of traditional cytogenetic analyses, making it accessible for all raised cattle rather than being limited to randomly sampled individuals due to the high cost of traditional cytogenetic analysis. By offering a low-cost alternative, the proposed method makes rob(1;29) translocation screening more accessible to a broader range of breeders and organizations involved in cattle management. The reduced financial burden associated with the analysis enables the implementation of widespread screening programs, aiding in the identification and management of rob(1;29) carriers within cattle populations. The cost-effectiveness of this approach further strengthens its potential as a valuable tool for mitigating the economic losses caused by translocation and preserving the genetic integrity of bovine breeds. An economic impact assessment has recently been published^21^: assuming a carrier bull frequency of 0.4% and a fertility decline of 5%, the elimination of carriers would lead to a savings of $2.8 million over 6 years. It should be noted that in some breeds, the frequency is much higher^22^.

One more advantage of this approach is the shorter response time, with the results obtained within a period of 12 h. Traditional cytogenetic analyses often require a waiting period of 7–10 days, which can significantly delay the identification of rob(1;29) carriers and subsequent actions for breeding programs. The quicker response time of the genomic biomarker allows for timely interventions and efficient management strategies, ensuring that carriers can be identified, and appropriate measures can be taken promptly. This expedited process minimizes waiting time, enhancing the overall efficiency and effectiveness of rob(1;29) translocation screening. It provides breeders and researchers with valuable insights into maintaining healthy and productive cattle populations.

To validate the efficacy of this method across all cattle breeds and biological matrices, the researchers of this study plan to expand their analysis to include a diverse range of breeds, encompassing both meat and milk purposes. By incorporating a broader spectrum of breeds, the robustness and applicability of the genomic biomarker can be ensured in detecting rob(1;29) translocation across different genetic backgrounds. This expanded analysis will provide comprehensive data on the accuracy and reliability of the biomarker, allowing assessment of its performance in various populations. This information will be crucial in determining its practicality and potential for widespread adoption among screening programs. Through an extensive validation process, the aim of this study was to establish the versatility and effectiveness of the genomic biomarker as a universal tool for the rapid identification of rob(1;29) carriers in all cattle breeds. Ultimately, this will contribute to managing cattle populations efficiently, minimizing economic losses, and safeguarding the genetic integrity of both meat and milk cattle breeds. In conclusion, the successful development of this genomic biomarker for the identification of rob(1;29) carriers opens up the possibility of transforming it into a marketable diagnostic kit. With its reliable and efficient performance, coupled with its advantages of applicability to various bovine biological matrices and low cost, this genomic biomarker has the potential to be commercialized as a user-friendly diagnostic tool. By packaging the necessary reagents and protocols into a comprehensive kit, it can be made readily available to cattle breeder’s associations and researchers. Such kits would enable widespread adoption and accessibility of the screening method, facilitating early identification of rob(1;29) carriers in different cattle breeds and assisting in effective breeding programs. The transfer of this technology into a marketable diagnostic kit would not only benefit the cattle industry by providing a practical and cost-effective solution but would also contribute to the enhancement of animal welfare and the management of their genetic value. Finally, it should be emphasized that this approach exploits its potential when a blood sample is not available (or it is complicated to get blood in good condition to the laboratory) and is not intended to replace the cytogenetic screening procedures currently in place. Over the years, these procedures have made it possible to identify numerous new abnormalities and eradicate them from populations at an early stage.

## Methods

### Whole genome sequencing

Whole-genome sequencing was performed using DNA extracted from the blood of a Maremmana subject who was homozygous for rob(1;29). A total of 500 ng of gDNA was sheared through sonication in 10 mM Tris–HCl, resulting in fragment sizes ranging from 100 to 1000 bp. DNA libraries were generated following standard protocol, including end repair, dA-tailing, adaptor ligation, and PCR enrichment (NEBNext® Ultra™ II DNA Library Prep Kit for Illumina®). The libraries were quantified using the Qubit™ dsDNA HS Assay Kit and analyzed with a High Sensitivity D1000 ScreenTape Assay kit on an Agilent TapeStation 4200 instrument. Subsequently, the libraries were sequenced using an Illumina HiSeq instrument with paired-end reads 150 bp in length. The FASTQ files were quality tested using FastQC (https://www.bioinformatics.babraham.ac.uk/projects/fastqc/).

### CNV analysis

The CNV search was performed manually using Integrative Genomics Viewers^23^.

### Study population

In this study, 94 cattle (Bos taurus) from seven different breeds were included (Table 2). All animals were previously tested for the rob(1;29) using standard cytogenetic analysis. Samples from Italian cattle breeds, which had been stored at − 20 °C for an extended period, were obtained from the animal cytogenetics laboratory at the University of Milan. Retinta samples, collected between 2007 and 2022 as part of the Retinta breeding program, were obtained from the molecular applied cytogenetic lab at the University of Córdoba, Spain. Genomic DNA was extracted directly from the blood samples using standard procedures (GenElute™ Mammalian Genomic DNA Miniprep Kits, Merck). The DNA yield and purity of each sample were measured using a NanoDrop ND-1000 spectrophotometer (Thermo Scientific). All samples used in this analysis met the following requirements: yield > 30 ng/μL, 260/280 ratio > 1.7, and 260/230 ratio > 1.8.

**Table 2:** Cattle breed Subjects analysed in population study, categorized as Wild type (Wt), Heterozygous carriers (HET), and Homozygous carriers (HOM).

Cattle breeds	Wt	HET	HOM	Tot
Agerolese	4	0	0	4
Maremmana	2	2	1	5
Chianina	1	1	0	2
Romagnola	1	12	0	13
Holstain Fresian	4	0	0	4
Marchigiana	2	10	0	12
Retinta	0	42	12	54
Total	14	67	13	94

All blood samples, including those in this study, were frozen and available for routine analysis in the laboratories, and no samples were specifically collected for this study; rather, these samples were collected over approximately 20 years of routine analysis.

For the study, ethical approval was waived, as the investigation did not involve an “animal experiment,” as defined by the exemptions outlined in Italian legislative decree n. 26/2014 (Dir. 2010/63/UE on the protection of animals used for scientific purposes.

All methods were carried out in accordance with relevant guidelines and regulation^24^. The q-PCR analysis was conducted using a 96-well CFX.

All methods employed in this study adhered to the guidelines of the RT-PCR System from Bio-Rad. Each reaction mixture consisted of a total volume of 10 μL, containing 30 ng of genomic DNA, iTaq Universal SYBR Green Supermix from Bio-Rad, 500 nM of the target (T) primer, 500 nM reference primer (defined as single copy gene-SCG), and water. Unfortunately, the primer design cannot be disclosed in this publication to ensure confidentiality associated with a pending patent application.

The thermal cycling profile followed the indications of Iannuzzi et al.^25,26^. This profile consisted of Stage 1:3 min at 95 °C; Stage 2:35 cycles of 15 s at 94 °C, 40 s at 55 °C, 30 s at 70 °C with signal acquisition, followed by 15 s at 83 °C with signal acquisition; and the final melt curve step (55–95 °C) with signal acquisition. The dissociation curve confirmed the specificity of both primers, as shown in Supplementary Fig. 3S. To ensure accuracy and reliability, each sample was processed in triplicate for both intra-assay and inter-assay analyses. In addition, a negative control (NTC) was included in each run. The standard deviation (SD) of the threshold cycles (Ct) was calculated for each sample. Subsequently, the samples were normalized using the ΔΔCt method with the assistance of CFX software.

### Statistical analysis

To evaluate the significance among different RNE values, statistical analyses were performed using the Kruskal–Wallis one-way ANOVA with Bonferroni correction. The minimum significance was set at p < 0.001.

### Supplementary Information

Supplementary Information.

## Data Availability

The datasets generated during and/or analysed during the current study are available from the corresponding author, AI, upon reasonable request.
