# Supplementary material for: A genomic biomarker for the rapid identification of the rob(1;29) translocation in beef cattle breeds
Source: Sci Rep. 2024 Feb 5;14:2951. doi: 10.1038/s41598-024-53232-8 (PMC10844278; doi:10.1038/s41598-024-53232-8)
Supplement: Supplementary file 1 — Supplementary Information. [file 41598_2024_53232_MOESM1_ESM.docx]

**Supplementary Information**

**Figure 1S**: Metaphases of rob(1;29) carrier subjects. **a.** Metaphase obtained for a female rob(1;29) carrier subjects; **b.** Metaphase obtained for a male rob(1;29) carrier subjects. Blue arrows indicate the sex chromosome; red arrows indicate the rob(1;29) chromosomes.


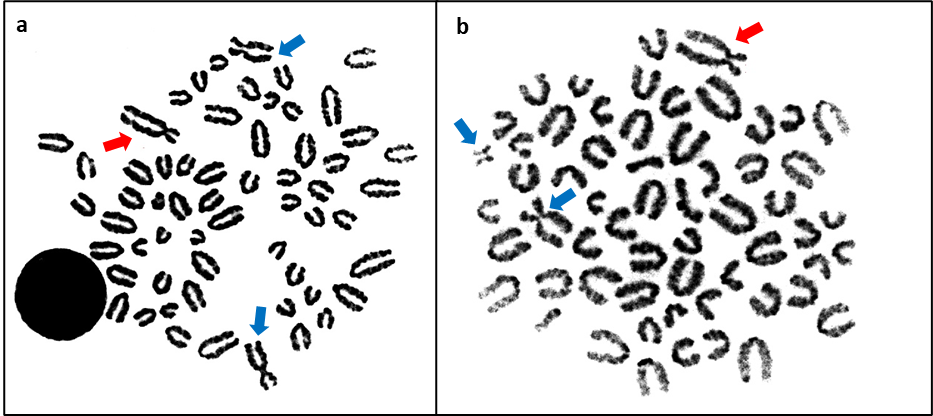


**Figure 2S**: Incidence of rob(1;29). The percentages of rob(1;29) carriers over the years are indicated in various years.


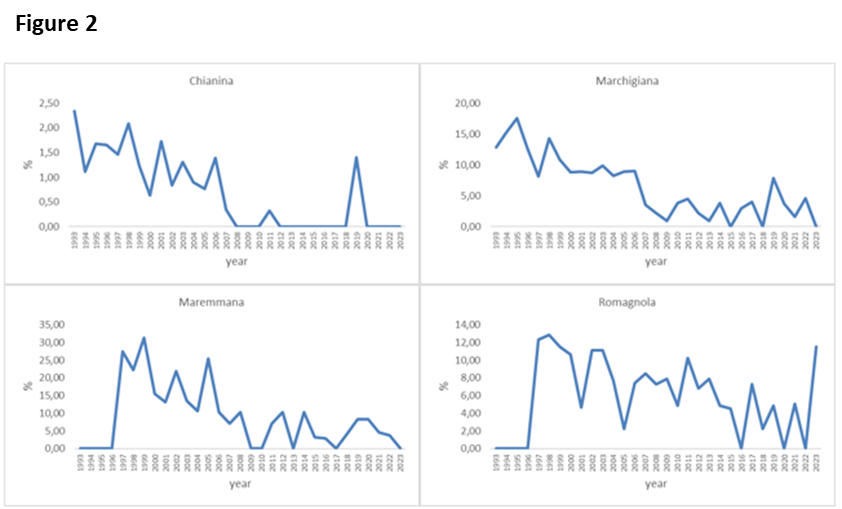


**Figure 3S: a.** Moving average (50 successive SNPs) of alternate variant frequencies.


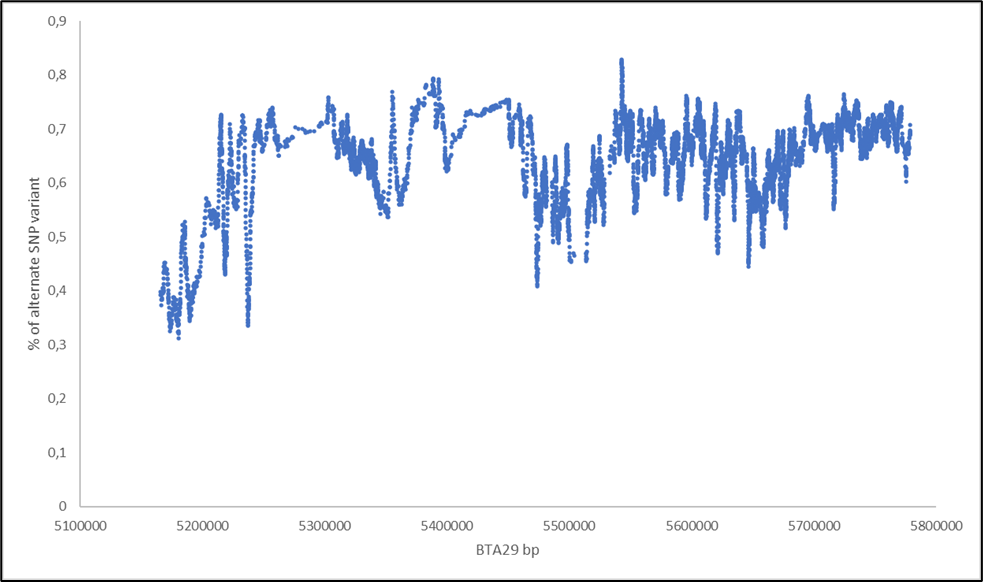


**Figure 4S: a.** Melting curves following 35 cycles of amplification (Thermal profile given in Materials and methods section) of 30 ng of bovine genomic DNA with target (t) and single-copy gene (scg) primers. The 77°C read provides the Ct value for the t primer, while the 88°C read provides the Ct value for the scg primer. The No template control (NTC) is represented in black; **b.** Bar Chart showing Relative Normalized Expression (RNE) of 16 cattle samples included in the analyses. Among these samples, two showed normal expression levels, exhibiting the lowest RNE. Ten samples were heterozygous carriers of rob (1;29), and four samples were homozygous carriers of rob (1;29) demonstrating the highest RNE.


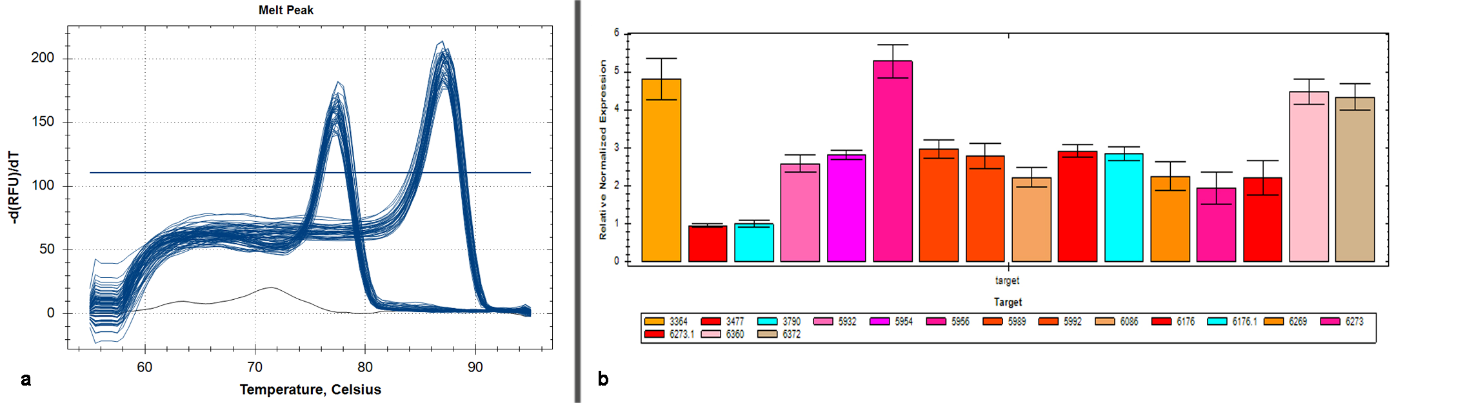


**Table 1S:** All beef cattle Subject analysed since 1993, and rob(1;29) carriers identified for each breed.

|  | **Chianina** | | | **Marchigiana** | | | **Maremmana** | | | **Romagnola** | | |
| --- | --- | --- | --- | --- | --- | --- | --- | --- | --- | --- | --- | --- |
| Year | **S.t.** | **rob(1;29)** | **%** | **S.t.** | **rob(1;29)** | **%** | **S.t.** | **rob(1;29)** | **%** | **S.t.** | **rob(1;29)** | **%** |
| 1993 | 1114 | 26 | 2,33 | 520 | 67 | 12,88 |  |  |  | 9 | 0 | 0,00 |
| 1994 | 987 | 11 | 1,11 | 493 | 75 | 15,21 |  |  |  |  |  |  |
| 1995 | 956 | 16 | 1,67 | 785 | 138 | 17,58 |  |  |  |  |  |  |
| 1996 | 1448 | 24 | 1,66 | 675 | 85 | 12,59 |  |  |  |  |  |  |
| 1997 | 341 | 5 | 1,47 | 220 | 18 | 8,18 | 109 | 30 | 27,52 | 178 | 22 | 12,36 |
| 1998 | 383 | 8 | 2,09 | 308 | 44 | 14,29 | 90 | 20 | 22,22 | 233 | 30 | 12,88 |
| 1999 | 325 | 4 | 1,23 | 296 | 32 | 10,81 | 99 | 31 | 31,31 | 200 | 23 | 11,50 |
| 2000 | 315 | 2 | 0,63 | 249 | 22 | 8,84 | 116 | 18 | 15,52 | 178 | 19 | 10,67 |
| 2001 | 289 | 5 | 1,73 | 224 | 20 | 8,93 | 38 | 5 | 13,16 | 171 | 8 | 4,68 |
| 2002 | 356 | 3 | 0,84 | 252 | 22 | 8,73 | 82 | 18 | 21,95 | 180 | 20 | 11,11 |
| 2003 | 383 | 5 | 1,31 | 284 | 28 | 9,86 | 81 | 11 | 13,58 | 144 | 16 | 11,11 |
| 2004 | 332 | 3 | 0,90 | 350 | 29 | 8,29 | 84 | 9 | 10,71 | 156 | 12 | 7,69 |
| 2005 | 394 | 3 | 0,76 | 314 | 28 | 8,92 | 130 | 33 | 25,38 | 132 | 3 | 2,27 |
| 2006 | 433 | 6 | 1,39 | 409 | 37 | 9,05 | 58 | 6 | 10,34 | 188 | 14 | 7,45 |
| 2007 | 292 | 1 | 0,34 | 228 | 8 | 3,51 | 71 | 5 | 7,04 | 129 | 11 | 8,53 |
| 2008 | 139 | 0 | 0,00 | 92 | 2 | 2,17 | 39 | 4 | 10,26 | 110 | 8 | 7,27 |
| 2009 | 161 | 0 | 0,00 | 104 | 1 | 0,96 |  |  |  | 127 | 10 | 7,87 |
| 2010 | 62 | 0 | 0,00 | 52 | 2 | 3,85 |  |  |  | 82 | 4 | 4,88 |
| 2011 | 308 | 1 | 0,32 | 266 | 12 | 4,51 | 71 | 5 | 7,04 | 137 | 14 | 10,22 |
| 2012 | 139 | 0 | 0,00 | 92 | 2 | 2,17 | 39 | 4 | 10,26 | 117 | 8 | 6,84 |
| 2013 | 161 | 0 | 0,00 | 104 | 1 | 0,96 |  |  |  | 127 | 10 | 7,87 |
| 2014 | 62 | 0 | 0,00 | 52 | 2 | 3,85 | 39 | 4 | 10,26 | 82 | 4 | 4,88 |
| 2015 | 104 | 0 | 0,00 | 87 | 0 | 0,00 | 30 | 1 | 3,33 | 110 | 5 | 4,55 |
| 2016 | 80 | 0 | 0,00 | 67 | 2 | 2,99 | 34 | 1 | 2,94 | 61 | 0 | 0,00 |
| 2017 | 28 | 0 | 0,00 | 50 | 2 | 4,00 |  |  |  | 41 | 3 | 7,32 |
| 2018 | 38 | 0 | 0,00 | 47 | 0 | 0,00 | 24 | 1 | 4,17 | 44 | 1 | 2,27 |
| 2019 | 71 | 1 | 1,41 | 64 | 5 | 7,81 | 24 | 2 | 8,33 | 62 | 3 | 4,84 |
| 2020 | 67 | 0 | 0,00 | 81 | 3 | 3,70 | 24 | 2 | 8,33 | 68 | 0 | 0,00 |
| 2021 | 59 | 0 | 0,00 | 61 | 1 | 1,64 | 44 | 2 | 4,55 | 59 | 3 | 5,08 |
| 2022 | 53 | 0 | 0,00 | 44 | 2 | 4,55 | 27 | 1 | 3,70 | 46 | 0 | 0,00 |
| 2023 | 30 | 0 | 0,00 | 31 | 0 | 0,00 |  |  |  | 26 | 3 | 11,54 |
| *Total* | *9910* | *124* | *1,25* | *6901* | *690* | *10,00* | *1353* | *213* | *15,74* | *3197* | *254* | *7,94* |

*S.t. Subject tested*

*rob(1;29)*

*% frequencies of rob(1;29) carrier subjects in different cattle breeds*

**Table S2**

Genomic elements present in CNV

Start Stop Gene symbol Strand

5.190.831 5.211.296 TRIM77 plus

5.228.004 5.234.027 ENSBTAG00000052850 plus

5.229.581 5.230.876 LOC100139585 plus

5.231.578 5.233.082 LOC107131949 plus

5.247.502 5.255.571 TRIM64 minus

5.308.698 5.308.806 LOC112444943 plus

5.311.985 5.317.066 ENSBTAG00000052563 plus

5.332.477 5.341.380 LOC112444837 minus

5.340.117 5.343.452 ENSBTAG00000046740 minus

5.368.066 5.370.588 LOC616911 plus

5.376.374 5.389.071 LOC101910072 plus

5.392.234 5.401.165 LOC521981 plus

5.394.617 5.399.905 ENSBTAG00000053004 plus

5.418.325 5.426.846 LOC100139381 minus

5.473.813 5.482.393 LOC788345 plus

5.497.084 5.498.340 LOC100337032 minus

5.536.728 5.539.338 LOC112444838 plus

5.537.527 5.539.730 ENSBTAG00000048894 plus

5.547.105 5.556.886 LOC530556 minus

5.556.596 5.556.937 ENSBTAG00000049159 minus

5.565.467 5.565.575 LOC112444942 minus

5.581.142 5.585.730 ENSBTAG00000051846 minus

5.584.472 5.585.715 LOC100138009 minus

5.614.048 5.623.542 LOC112444922 minus

5.644.344 5.648.725 LOC786425 plus

5.653.275 5.667.685 ENSBTAG00000054977 minus

5.684.573 5.686.105 TRIM48 plus

5.714.056 5.722.971 LOC786377 plus

5.747.175 5.748.648 LOC523762 plus

5.747.233 5.748.446 ENSBTAG00000051538 plus

5.755.909 5.776.998 LOC100336475 plus

5.765.565 5.765.673 LOC112444941 plus

5.774.213 5.774.554 ENSBTAG00000048802 plus
